# A prospective study of *Trichomonas vaginalis* and prostate cancer risk among African American men

**DOI:** 10.1186/s13104-016-2033-3

**Published:** 2016-04-18

**Authors:** Jay H. Fowke, Xijing Han, J. F. Alderete, Kelvin A. Moses, Lisa B. Signorello, William J. Blot

**Affiliations:** Departments of Medicine, Institute of Medicine and Public Health, Vanderbilt University Medical Center, 2525 West End Ave, 12th floor, Nashville, TN 37232 USA; International Epidemiology Institute, Rockville, MD USA; School of Molecular Biosciences, Washington State University, Pullman, WA USA; Department of Urologic Surgery, Vanderbilt University Medical Center, Nashville, USA; Division of Cancer Prevention, National Cancer Institute, National Institutes of Health, Bethesda, MD USA

**Keywords:** Prostate cancer, *Trichomonas vaginalis*, Race

## Abstract

**Background:**

African Americans (AA) have a higher prevalence of *Trichomonas vaginalis* (*Tv*) infection and a higher prostate (PC) risk. Past studies suggest an association between *Tv* seropositivity and PC, and therefore we prospectively investigated this association among AA men.

**Results:**

Incident PC cases were individually matched to controls in a nested case–control study within the Southern Community Cohort Study (SCCS). Primary analysis included 296 PC cases and 497 race-matched controls. Levels of *Tv* antibody response were measured by ELISA in serum collected at baseline. *Tv* antibody response did not significantly differ between cases and controls overall or within AA participants (253 AA cases). There were no significant associations or trends between levels of *Tv* response and PC risk or the diagnosis of aggressive PC.

**Conclusion:**

We found no evidence of a prospective association between baseline *Tv* infection and PC risk in AA men. *Tv* infection in men may have substantial health implications in HIV transmission and reproductive outcomes, but may not impact future PC risk in AA men at high-risk for PC. Further efforts need to define past vs. present *Tv* infection and to separate pathophysiology from PC detection.

## Background

*Trichomonas* vaginalis (*Tv*) is a sexually transmitted protozoan parasite. In men, urethral infection may ascend to the prostatic urethra and glandular tissue. With asymptomatic infection common, *Tv* exposure may induce a sustained inflammatory response to advance prostate carcinogenesis [1]. Analysis of the Health Professionals Follow-up Study and the Physicians Health Study found that *Tv* antibody seropositivity was significantly associated with prostate cancer (PC) risk [2, 3] or PC death [2]. In contrast, *Tv* seropositivity was not associated with PC risk in the Prostate Cancer Prevention Trial (PCPT) [4] or a population-based case–control analysis [5]. The purpose of this study was to determine the prospective relationship between *Tv* infection and PC risk among African American (AA) men. Past studies included few AA men, although there are data suggesting the prevalence of *Tv* infection and the risk of PC may be greater than among white men [6].

## Methods

Details of the Southern Community Cohort Study (SCCS) have been published [7]. Incident PC cases among nearly 35,000 male participants enrolled at age 40–79 during 2002–2009 were identified through linkages through 2013 with state tumor registries and the National Death Index. All participants provided written informed consent, and all protocols were approved by Institutional Review Boards at Vanderbilt University and Meharry Medical College. All SCCS data can be requested through an online request (southerncommunitystudy.org). The request will be reviewed by the SCCS Data and Biospecimen Use Committee to ensure that it is scientifically justified and that participant confidentiality is preserved. Two controls per case were selected by incidence density sampling and were individually matched by age (5 years), race (self-reported black or white), site, and time of donation of blood samples at study entry. A blood sample was collected at baseline recruitment, and serum was frozen-stored at −80 ^°^C. Serum was assayed in duplicate for antibodies against *Tv* by ELISA to detect the IgG antibodies against the purified recombinant *Tv* α-actinin protein and assessed spectrophotometrically [8]. We previously assigned scores from 0 to 4+ to serum based on the calculation of P/N values obtained using the protein ACT-P2 as the target [2–4, 8]. For this study, cutoff points for seropositivity were obtained by dividing the average OD_405nm_ of the seropositive control serum by the corresponding seronegative control serum. The lowest seronegative control was assigned a score of 0 (zero). Values from 0 to the next lowest was given a score of 1+, and subsequent scores of 2+, 3+, and 4+ were assigned similarly as values increased. The sera with P/N scores ≤2 were negative as evidenced by lack of detection of any *T. vaginalis* proteins by immunoblot [8]. Scores ≥3 were positive and had antibody to α-actinin and other trichomonad proteins [8]. We used conditional logistic regression to compute odds ratios (OR) and 95 % confidence intervals while controlling for household income. Cancer stage or Gleason score from state tumor registries providing these data were also analyzed after controlling for age, race, and income.

## Results

The majority of the study population was over 60 years of age at recruitment (n = 549, 62 %), and reported a household income less than $15,000/year (n = 512, 58 %). Approximately 85 % of the study population was AA. Table 1 summarizes the association between *Tv* seropositive status and PC. Mean antibody response levels were similar between cases and controls (all p > 0.05). There was no significant association between *Tv* and PC in the total study population, or when restricting to AA men. Furthermore, *Tv* exposure was not associated with PC Gleason score of 7 or more, or stage 2–4 PC at diagnosis, in a case-only analysis (Table 2).

**Table 1:** *Trichomonas vaginalis* and association with prostate cancer risk

*T. vaginalis*	Cases	Controls	OR	95 % CI
n	296	585		
Mean (SD)	0.26 (0.11)	0.26 (0.11)		
Serostatus				
Negative	227 (76.7 %)	461 (78.8 %)	1.0	Ref
Positive	69 (23.3 %)	124 (21.2 %)	1.11	0.77–1.61
Score				
0	60 (20.3 %)	132 (22.6 %)	1.0	Ref
1	95 (32.1 %)	168 (28.7 %)	1.18	0.77, 1.82
2	72 (24.3 %)	161 (27.5 %)	0.94	0.58–1.53
3	50 (16.9 %)	84 (14.4 %)	1.25	0.74–2.11
4+	19 (6.4 %)	40 (6.8 %)	0.98	0.48–1.97
African Americans Only^a^				
n	253	497		
Mean (SD)	0.26 (0.11)	0.26 (0.11)		
Serostatus				
Neg	191 (75.5 %)	386 (77.7 %)	1.0	Ref
Pos	62 (24.5 %)	111 (22.3 %)	1.12	0.76–1.66
Score				
0	50 (19.8 %)	104 (20.9 %)	1.0	Ref
1	80 (31.6 %)	145 (29.2 %)	1.10	0.69–1.76
2	61 (24.1 %)	137 (27.6 %)	0.87	0.51–1.47
3	44 (17.4 %)	74 (14.9 %)	1.18	0.67–2.08
4+	18 (7.1 %)	37 (7.4 %)	0.95	0.45–1.97

Adjusted for age at diagnosis, race, income

^a^Adjusted for age at diagnosis and income*

**Table 2:** *Trichomonas vaginalis* seropositive status and diagnosis of aggressive prostate cancer

Diagnosis of aggressive PC^a^				
	Gleason < 7	Gleason ≥ 7	OR	95 % CI
n	74	101		
Mean (SD)	0.25 (0.10)	0.26 (0.10)		
Serostatus				
Negative	60 (81.1 %)	80 (79.2 %)		
Positive	14 (18.9 %)	21 (20.8 %)	1.04	0.48–2.26

	Stage 0–1	Stage 2–4		
n	230	40		
Mean (SD)	0.25 (0.12)	0.26 (0.10)		
Serostatus				
Negative	179 (77.8 %)	30 (75.0 %)		
Positive	51 (22.2 %)	10 (25.0 %)	1.23	0.55–2.74

Stage and grade data were not provided by every tumor registry

^a^Adjusted for age at diagnosis, race, and income

## Discussion

*Tv* disproportionately affects minorities and low-income populations [6]. However, unlike two past studies of predominately white professionals [2, 3], we found no evidence of association within AA men. A post hoc power calculation based on a similar matched nested case–control design and assuming a 25 % exposure, Type I error of 5 %, and 80 % power, would be able to detect an OR = 1.42, suggesting a sufficient sample size to identify a moderate association. Residual confounding is unlikely to have led to a null association. As previously detailed, differences in patient characteristics and detection protocols could explain differences in results across studies [4]. Similarly, an inflammatory effect from *Tv* would likely increase PSA levels, and thus increase the likelihood of detecting an asymptomatic PC and lead to a positive association. No association between *Tv* seropositivity and PC was found in the PCPT, with rigorous control for PC screening including an end of study prostate biopsy [4]. Alternatively, analysis of the Prostate, Lung, Colorectal and Ovarian cancer screening trial found *Tv* seropositivity significantly associated with benign prostatic hyperplasia [9], suggesting *Tv* may exacerbate lower urinary tract symptom severity and possibly lead to a PC detection. [10] *Tv* seropositivity is relatively stable over time [10], limiting the ability to identify an etiologically relevant time of exposure. Our prospective analysis found no association between baseline Tv seropositivity and future PC risk, and further efforts are needed to define past vs. recent *Tv* infection and to separate pathophysiology from the detection of PC.

## Abbreviations

AA: African American
CI: confidence interval
OR: odds ratio
PC: prostate cancer
PCPT: prostate cancer prevention trial
P/N: positive/negative ratio
SCCS: Southern Community Cohort Study
Tv: *Trichomonas vaginalis*
